# Optimization of solid waste collection using RSM approach, and strategies delivering sustainable development goals (SDG’s) in Jeddah, Saudi Arabia

**DOI:** 10.1038/s41598-021-96210-0

**Published:** 2021-08-16

**Authors:** Neyara Radwan, Nadeem A. Khan, Rania Abdou Gaber Elmanfaloty

**Affiliations:** 1 Faculty of Economics & Administration, King Abdulaziz University, Jeddah, 21589 Saudi Arabia; 2 Mechanical Department, Faculty of Engineering, Suez Canal University, Ismailia, Egypt; 3 Department of Civil Engineering, Jamia Millia Islamia, New Delhi, 110025 India; 4 Department of Electrical and Computer Engineering, Faculty of Engineering, King Abdulaziz University, Jeddah, 21589 Saudi Arabia; 5 Department of Electronics and Communications Engineering, Alexandria Higher Institute of Engineering and Technology, Alexandria, Egypt

**Keywords:** Environmental impact, Environmental impact, Climate sciences, Environmental sciences, Environmental social sciences, Energy science and technology, Engineering

## Abstract

The rapid industrial development, high population growth, and rapid urbanization of Saudi Arabia have led to increased pollution and waste levels. Every day, solid waste disposal for governments and local authorities becomes a significant challenge. Saudi Arabia produces over 15 million tonnes of solid waste annually, with a population of around 29 million. The waste production per person is estimated at between 1.5 and 1.8 kg per day per person. About 75% of the population in urban areas is concentrated, making it imperative that government steps are taken to boost the country's waste recycling and management scenario. The production of solid waste in Riyadh, Jeddah, and Dammam, three of the largest cities, exceeds seven million tonnes annually, which shows the enormity of the civic body problem. During this study, the design Expert software was involved in the optimization of process parameters during the collection of municipal solid waste (MSW) from Jeddah city. The use of design experiments and numerical optimization is quite effective in optimizing the different process parameters on the overall cost. Saudi Arabia has a critical need for a resilient waste system and agile waste management system to control its municipal solid waste quickly and environmentally friendly for achieve Saudi Vision 2030. For this study design of experiment, software was employed to optimize the cost per trip, thereby considering process parameters. It is therefore essential to examine the existing practices and future opportunities for solid waste collection, storage, and disposal. This study considered that MSW generated in Saudi Arabia which is having great potential to be converted into wealth. Hence, considering the current environment situation, energy prospective and future management strategies for MSW have also been reviewed.

## Introduction

### General background

**Figure 1:**
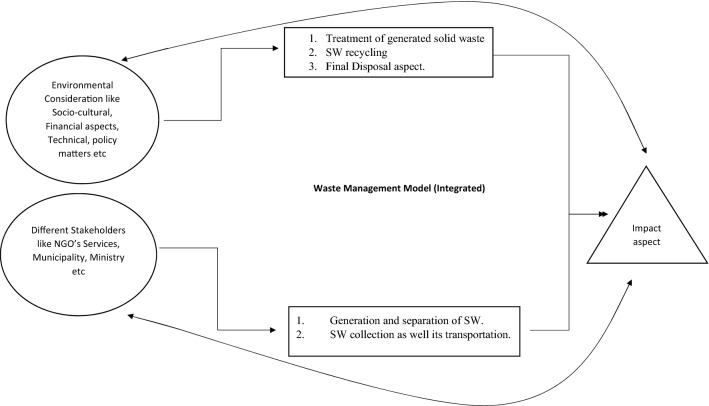
Management of solid waste using integrated management model^5^.

The population rise, economic growth that has rapidly accelerated urbanization, and changed public living standards have resulted in an increase in municipal solid waste (MSW) production in India^1^. The amount of waste is estimated at 64–72 million tonnes at present and is estimated to rise to around 125 million tonnes by 2031^2^. The compilation of household and commercial waste by public-produced sources is usually MSW^3^. Generally speaking, MSW includes paper and sheets of paper, food, textile, wood waste, and fully non-degradable goods such as leather, rubber, metal, and glass^4^. The shown Fig. 1 illustrates the concept of waste and integrated management model^5^. Disposals of urban solid waste constantly and carelessly conducted regularly contribute to a wrong way of life and lack of awareness of the environment^6^. In addition, ineffective processes of waste collection and insufficient transport facilities are mainly responsible for the accretion of MSW throughout the city^7^. Non-science monitoring and disposal of MSW are responsible for numerous complications linked to environmental contamination and public health and well-being problems^8^. It is essential for professional and efficient MSW management for enhanced and secure lifestyles and sustainable development that the characterization and proper collection and storage schemes, waste transport systems, and final dumping are identified^9^. Several researchers have established factors affecting the waste management system's components^10^. The amount of waste produced is determined by the size of the household, their level of schooling, and their monthly income^11^.

Household views toward waste separation are influenced by a real estate company's constructive sponsorship and commitment, the role of neighbourhood residential councils in facilitating civic interaction, and a fee for recycling service dependent on waste volume or weight^12^. Gender, peer control, the size of the land on which the household resides, the location of the household, and membership in an environmental association all contribute to household waste use and separation behaviour^13^.

Waste disposal remains largely spontaneous and unregulated in developing countries. The collection services of MSW face a growing number of problems in the Kingdom of Saudi Arabia, such as grower population, variations in customary conditions, and a lack of awareness of the environmental effects of solid waste^13^. Therefore, this paper aims to review the present MSW in Jeddah besides recommending appropriate management practices toward improvement in MSW management, which would eventually improve environmental security^14^. One of the main issues adopted by the League of Arab States in its plans for 2008 was solid waste disposal^15^. The league calls for researchers to draw up projects on solid waste management for the Arab region at the national level^16^. In addition, an integrated hazardous material and waste management plan should be placed in place. MSW poses a risk to residents due to their critical environmental pollution role^17^. The proper management of solid waste (such as processed, collected, disposed of, and recycled) requires detailed information about the rate and quantity, composition, source, and location of waste generated^18^. In light of the COVID-19 scourge, this article demonstrates that extensive analysis is needed to assess the business as usual approach to plague mindfulness and response in biomedical wastes^4,18^. We must expose the debate around future improvements to practise, for the sorting, collecting as well as handling of biological waste products from clinics and isolation facilities of positive or suspicious COVID-19 patients^4,18–22^.

Although the latest procedures, like the retaining periods, might significantly reduce the risks to laborers involved in the hazardous wastes management, the latest device modifications may be needed and should be considered^2^. Additionally, urgent need for knowledge on SARS-CoV-2 prevalence and detection in biomedical waste to comprehend associated dissemination mechanisms and to illuminate proper risk prediction and management practices for the biomedical sector^21^. As per the latest investigation into a possible airborne transmission of COVID-19 is also a major concern, including SARS-CoV, shown that this could be a prospective route in disease dissemination^23^. The facility to segregate SARS-CoV-2 from medical waste provides an ideal prospect to reintroduce its value as a statistics source^2^. Also, unique safety measures and the need to ensure information security can present a trial, given the opportunity, human expense, and commercial impact of COVID-19, such investigation should proceed with zeal^7^. The cities of Jeddah in Saudi Arabia do not have this type of information^24^. There are several active case studies on the involvement of the local and private sectors in the management of solid waste in developing countries.

Saudi Arabia has a critical need for a resilient waste system and agile waste management system to control its municipal solid waste quickly and environmentally friendly. For this study design of experiment, software was employed to optimize the cost per trip, thereby considering process parameters. It is therefore essential to examine the existing practices and future opportunities for solid waste collection, storage, and disposal. During this study, the design expert software was involved in the optimization of process parameters during the collection of MSW from Jeddah city. The use of design experiments and numerical optimization is quite effective in optimizing the different process parameters on the overall cost. This study considered that MSW generated in Saudi Arabia which is having great potential to be converted into wealth. Hence, considering the current environment situation, energy prospective and future management strategies for MSW have also been reviewed. This study considered that MSW generated in Saudi Arabia which is having great potential to be converted into wealth^25,26^. Hence, considering the current environment situation, energy prospective and future management strategies for MSW have been also reviewed in this paper.

### MSW management system used in Saudi Arabia

In contrast to other infrastructure services, the literature on performance evaluation of multi-criteria decision-making (MCDM) systems stays rarer. This needed to be supported by a comprehensive literature review of MSW systems performance evaluation using different models. Some related studies are listed here briefly. Recently conducted a systematic analysis of various MCDM applications in waste management^27^. The study shows that most studies discussed problem selection of the locations of the sites, social and environmental impact assessments of various waste management technology, and the selection of waste processing plant types^27^. In regions with a robust seasonal tourism presence in Portugal, Mendes et al. have introduced waste management systems using a simple weighted sum method for testing their performance. It considered 39 municipal waste systems in Portugal based on a simple cross-comparison. For the performance enhancement of waste management programs, the driver pressure-state impact-response model used eighteen performance indicators (PI). The output (response) for each PI was assessed with the simple addition^28^.

The European Union (EU) participated in the waste sector by regulations that promoted independent curbside recycling of recyclables and residual waste. Additionally, while the EU has shifted its emphasis away from waste dumping and toward recycling and reuse, the focus in low-income countries around the world has been on expanding service reach^29^. Given the scarcity of resources affecting the public sector and the tightening budget constraints imposed by EU agreements, municipalities can pursue one of three strategies to raise funds necessary to meet higher quality standards:

1. increase taxes and tariffs;
2. increase borrowing from banks and private investors, thereby increasing debt; or
3. improve efficiency.

The first choice is very unpopular and contradicts lawmakers' need to be re-elected; additionally, it can be pursued only with the approval of city governments and the concession contracts that control waste facilities on a local level^6,7^. The performance of a particular component was evaluated in several of these studies using a collection of several PIs for regional/global comparisons from the raw data taken into account from many municipalities^15,30,31^. In 40 municipalities in Verona, Italy, Guerrini et al. measured their performance by non-parametrical methods focused on significant data parameters^29^. Anestina et al. analysed 30 businesses that have implemented the non-government sector contribution towards solid waste management in Nigeria for enhancing economic productivity and efficiency^29,32^. In Taiwan, Huang et al. conducted just five key measures in performance evaluations for 307 MSWM systems^33^. Lohri et al. assessed the financial viability of MSWM systems in Ethiopia using cost-income analysis. Abbondanza et al. have recently calculated the Brazilian e-waste generation^34^.

In past research, a specific feature of the MSWM system was protected with fewer PIs. A significant number of participating municipalities used detailed information sets of the past results^35^. Moreover, there were no considerations of uncertainties due to observational errors or restricted data. The first challenge is identifying a benchmark for evaluating the performance of MSWM systems in KSA and other similar countries^36^. Benchmarks can be defined as ideal benchmarks for quantitative PIs that have to be comparable or inferior to such standard values: noise levels, water quality, air quality, etc. Without prior information, the issue is more severe for qualitative PIs or newly formed PIs^37^. On the other hand, in order to establish priority based on individual experiences and expertise, different types of literature show uncertainty towards this approach.

### Objectives of the study and research gap

The purpose of this investigation was to examine and optimize the MSW cost per trip of Jeddah using experiment software. Furthermore, analysis of the basic composition of the solid waste in the region; treatment and management strategies; and investigation reuse options. The results obtained may be helpful in the strategic management of the MSW management program for reducing the risk in the Environment of Saudi Arabia.

This research will help to ascertain the effectiveness of a novel natural coagulant, Pricralima nitida extract (PNE), on municipal solid waste landfill leachate (MSWL) treatment before disposal through the bio-coagulation-flocculation (BCF) method. The main goal of this research was (i) to evaluate the applicability and performance of Picralima nitida extracts for the treatment of municipal-solid-waste leachate (MSWL) via the coagulation-flocculation process via, (ii) to model and optimize the process using RSM.

The proper management of solid waste (i.e., storage, collection, disposal, and recycling) requires accurate information on waste generation rates, quantities, composition, sources, and waste locations^38^. The towns of Jeddah, Saudi Arabia, cannot receive such information. There have been a number of successful case studies of participation by the community and private sectors in Solid Waste Management in developing countries^39^. Findings on waste management in different countries were presented in different studies but unfortunately, Jeddah does not carry out such case studies^40^. The present system for collecting and storing solid waste in Jeddah is also traditional and inefficient because various waste sources are combined. In addition, the amount of waste collected and the per capita waste generated by each individual person is not indicated in any sound record^1,4,11^. In this study, the characteristics, generation, collection, and transport of waste, disposal, and processing technologies practiced in Jeddah are examined comprehensively. A solid waste planning will be proposed on the basis of the results. The municipal public work services are carried out under municipal supervision by private contractors^8,13,14,18,19^. In a variety of containers, all municipal solid waste, including litter, is collected. The waste is often packaged before storage in the container in old shopping plastic bags and is sometimes dispersed bulk or bagged into trash bags^9,12,33^. This kind of disposal contributes substantially to the mixing of waste in storage containers on the spot^15,24,29^.

## Methodology

### Site description

**Figure 2:**
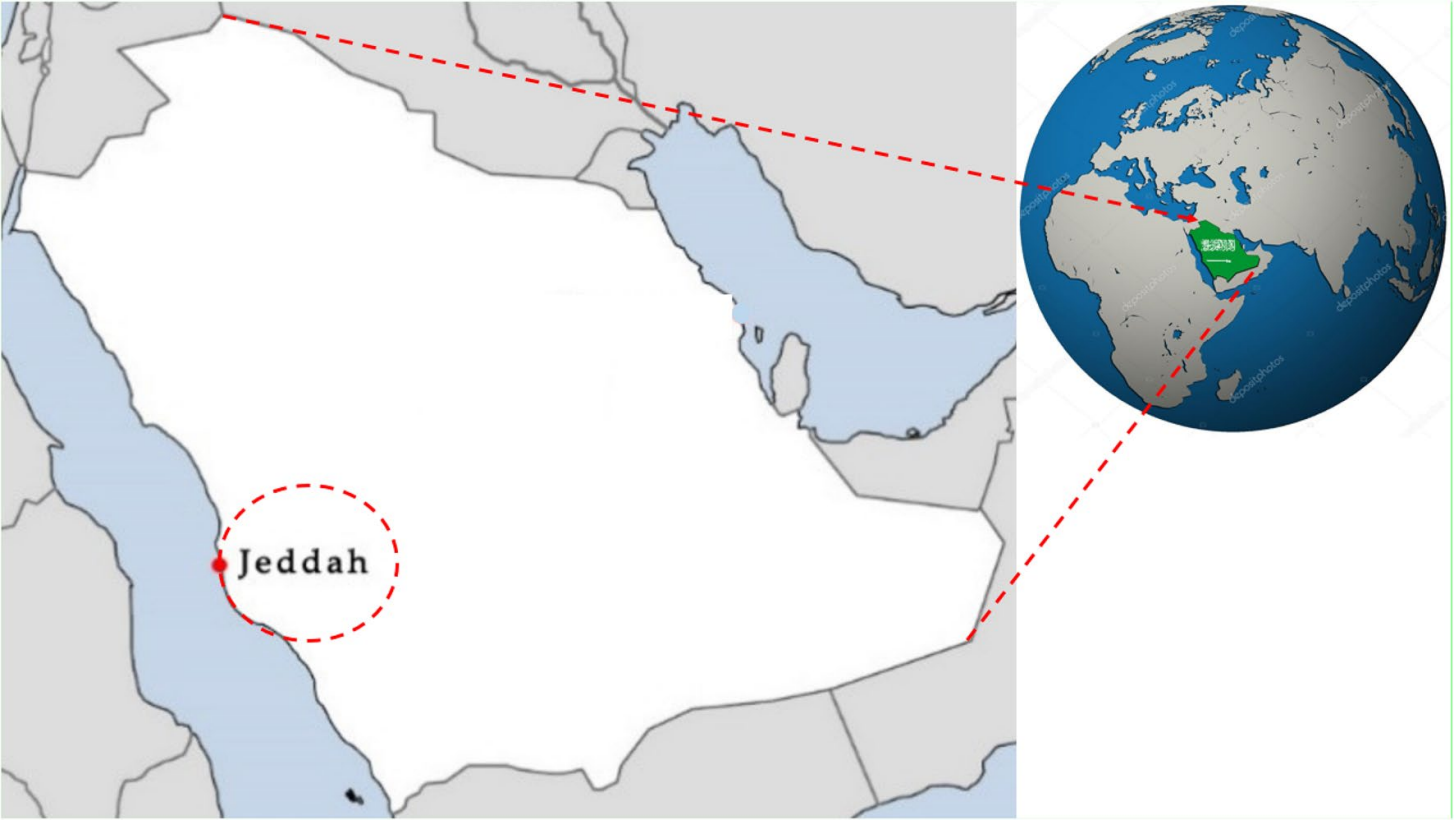
Location of Jeddah Region's selected collection and transfer stations (Satellite imagery source: The National Map; http://viewer.nationalmap.gov/viewer/).

The municipality of Jeddah city is located at Hejaz region of Saudi Arabia (21° 32′ 36″ N–39° 10′ 22″ E) and is the commercial center covering almost 1600 km^2^ city area. A population density of about 2500/km^2^ was reported in 2014. The population density is high, leading to compacted localities. The production of MSW in the selected city was around 118,190 tons per year, having daily production of around 1.24 kg/per/day. The municipality of Jeddah ensures around 162,000 household collection contracts with around 212 vehicles with a capacity of about 10–12 m^3^ to be collected in the mixed waste form. The MSW composition was calculated by collecting samples randomly from grids in Al-Ahsa, as shown in Fig. 2.

### SW collection process and sampling

This study was carried out for Jeddah city, Saudi Arabia, in which solid waste sample collection was observed for 254 days. In this study, the main-set parameters were considered to mix solid waste during and after collection; however, they are taken during the source separation of materials as per the data collected, and different analysis procedures are kept to be identical for the study. The collection was carried out from 80 different grids having drop-off (4 m^3^, 20 grids) or collection done at street-side bins (0.5 m^3^, 60 grids), having variable collection frequencies (weekly /or daily), and the system was set such that the activity done during 0–5 h, 5–10 h, 10–15 h, 15–20 h, 20–24 h. It is worth noting that all the vehicles employed having two workers from Sunday to Friday, 8 h of daily duty. Each vehicle crew performs duties as per the assigned grid by taking care of particular assigned containers or until complete vehicle volume is reached. This infers that the vehicle will reach up to the disposal site to upload the collected waste, traveling from disposal to parking station or likewise. Finally, the crew will return to parking and other purposes like lunch/refiling, etc. During a particular day, when data collection was taken into consideration, a sample of different crews/grid/route plan and time was taken. This observation was used to record manually by the vehicle crew particularly assigned to it, thereby gathering information related to the comprehensive waste collection cycle during a particular grid. This observation data collection includes vehicle number registration, grids allotted, container type, the total volume of solid waste collected, the volume of fuel consumed during the visit, distance as well as time of start and end. After looking at the raw data, removing the errors like missing data, duration or route plan, etc., a total of around 2512 observations were recorded. A few significant parameters were assessed in areas like the Medina MSW stream: *MSW generation rates, per capita amount of more MSW produced daily, and the composition of MSW*. The generation rate of MSW is determined by measuring the amount for the disposal of waste, the number, and the capability of each vehicle selection route. The mass production rate was calculated and divided by population. The MSW produced per capita was calculated.

The sectors selected include high-income, low income, and trade. Samples were subsequently categorized and weighed per category: Food waste, paper & cardboard, Glass, Plastic, Aluminum, Scrap metal, excluding aluminum, Rubber, Wood, Agricultural waste, Hazardous waste, and others such as electrical equipment, used furniture, electricity, etc.

Waste management is commonly used to mitigate the harmful health, the atmosphere, or the aesthetic impact of waste. Waste management includes all waste resource recycling operations. Waste management includes managing substances with minimal harmful consequences of solid, liquid, gaseous or non-hazardous wastes. Waste management means avoiding waste production, reducing waste volumes and their detrimental environmental effects, reducing waste volumes, or destroying nature. The main groups of waste are illustrated in Supplementary Fig. 2. Due to the density, lifestyle, and economic disparities, Jeddah City has been divided into five sample areas (north (N), north-central sampling area (CN); south-central sampling zone (CS); South (S) have shown in Supplementary Fig. 1.

Primarily it was essential to see that management of domestic MSW begins from processing facilities where this refuse is processed and divided according to hereditary form into colored containers (organics, paper, plastics, metals, and others). This move was essential to support and reduce waste management financial costs. The householders have to take time and effort to sort waste into a variety of tanks (typically recyclable waste and general waste), and as such, they are paid non-monetary costs. However, recycling systems that allow households not only to separate recyclables into paper, plastics, glass, or metals but to put many recyclable materials into one bin tend to generate far higher overall recyclable yields.

The rate of waste production and collection varies according to seasons and for other reasons. This may have little effect in incinerators of a batch type, but it certainly creates operational difficulties in incinerators of continuous type, especially when the inventory is not sufficient. Continuous incineration requires continuous supply to work efficiently. Any batch type of continuous form is also a gasifier. However, as the volumetric feed flow in gasification systems is much lower, continuity or humidity of feed flow is not a significant problem. The continuity of the MSW supply has a minor effect on composting.

### Optimization using RSM method

The optimization was done to minimize the cost of MSW collection in Jeddah city using design experiment software. The design expert software v.12 helps us to optimize the operation parameters using central composite design space. For this study, we have taken a two-level factorial design for this purpose, having 30 design runs. The design parameters under consideration are shown in Table 1.

**Table 1:** Process parameters used in the design of experiment.

S. No	Variable type	Coding	Coding range
1	No. of trips	A	10–40
2	Manpower	B	1–2
3	Fuel consumption (l)	C	10–30
4	Solid waste volume (m^3^)	D	2–6

It has been observed from the design expert analysis that F-value came out to be 61.89, implying that the model fits well for given parameters. The values of about 0.01% values might be due to noise, and P-value came out to be 0.05 shows model parameters are significant. In this model, the values of B, C, AB, AC, AD, BC, BD, CD, A^2^, B^2^, C^2^, D^2^ are seen to be significant during the analysis, as shown in Supplementary Tables 1 and 2. These values, as shown in Table 1, indicated model terms are not significant and can be left during analysis. The analysis of lack of fit for the given model having F-value seen to be 1.06 implies model terms are not significant compared to relative error. It is also to note that 50.49% chances are due to large noise values. The model seems to be fit well.

**Table 2:** Predicted and observed values.

S.No	Variable	Optimized value	Observed value
1	No. of trips	22	21
2	Manpower	2	2
3	Fuel consumption (l)	16	18
4	Solid waste volume (m^3^)	4	3.8

The Predicted R^2^ for the given set of parameters seen to be 0.9255, which is reasonable agreement with the Adjusted R^2^ of 0.9671; i.e., the difference is less than 0.2. The value of Adeq Precision measures the signal-to-noise ratio from the set of parameters. A ratio greater than 4 is seen to be desirable, as shown in Supplementary Table 3. Your ratio of 25.983 indicates an adequate signal. This model can be used to navigate the design space.

The coefficient estimate represents the expected change in response per unit change in factor value when all remaining factors are held constant. The intercept in an orthogonal design is the overall average response of all the runs. The coefficients are adjustments around that average based on the factor settings. When the factors are orthogonal, the VIFs are 1; VIFs greater than 1 indicate multicollinearity; the higher the VIF, the more severe the correlation of factors. As a rough rule, VIFs less than 10 are tolerable.

$$\begin{aligned} & {\text{Cost}}\,{\text{of}}\,{\text{solid}}\,{\text{waste}}\,{\text{estimated}}\,{\text{using}}\,{\text{RSM}}\,{\text{model}} \\ & \quad = {117} + 0.{2}0{8333}*{\text{A}} + - {1}.{2}0{833}*{\text{B}} + {1}.{625}*{\text{C}} + 0.{375}*{\text{D}} + - {2}.{3125}*{\text{AB}} \\ & \quad \quad + - 1.0625*{\text{AC}} + 1.8125*{\text{AD}} + - 3.5625*{\text{BC}} + - 2.1875*{\text{BD}} \\ & \quad \quad + - 0.9375*{\text{CD}} + 1.76042*{\text{A}}^{2} + 0.885417*{\text{B}}^{2} + 1.63542*{\text{C}}^{2} \\ & \quad \quad + 4.13542*{\text{D}}^{2} \\ \end{aligned}$$

The equation in terms of coded factors can be used to make predictions about the response for given levels of each factor. By default, the high levels of the factors are coded as + 1, and the low levels are coded as -1. The coded equation is useful for identifying the relative impact of the factors by comparing the factor coefficients.

**Figure 3:**
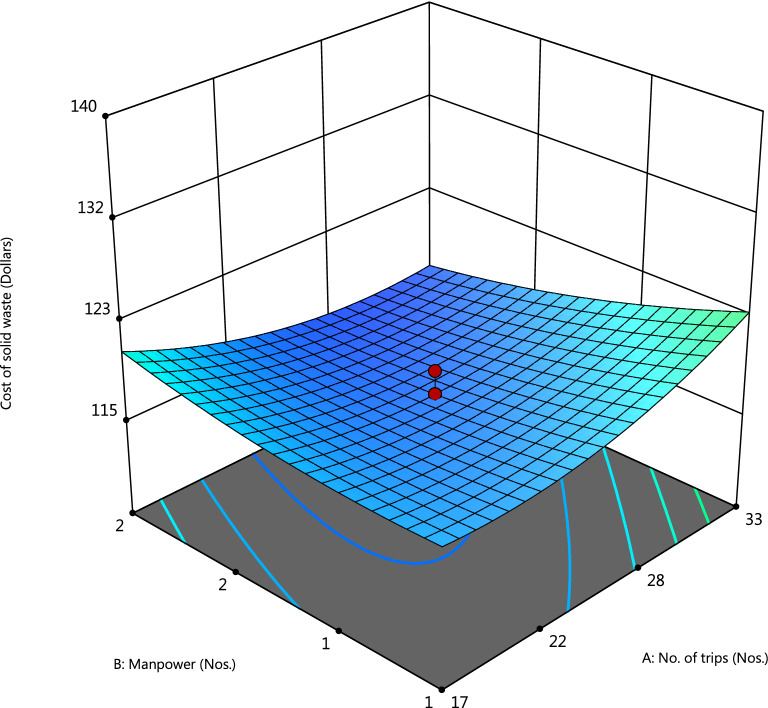
3D surface plots showing the dependency of the number of workers and number of collection trips on the cost of a solid waste management system (Design-Expert software v. 12).

**Figure 4:**
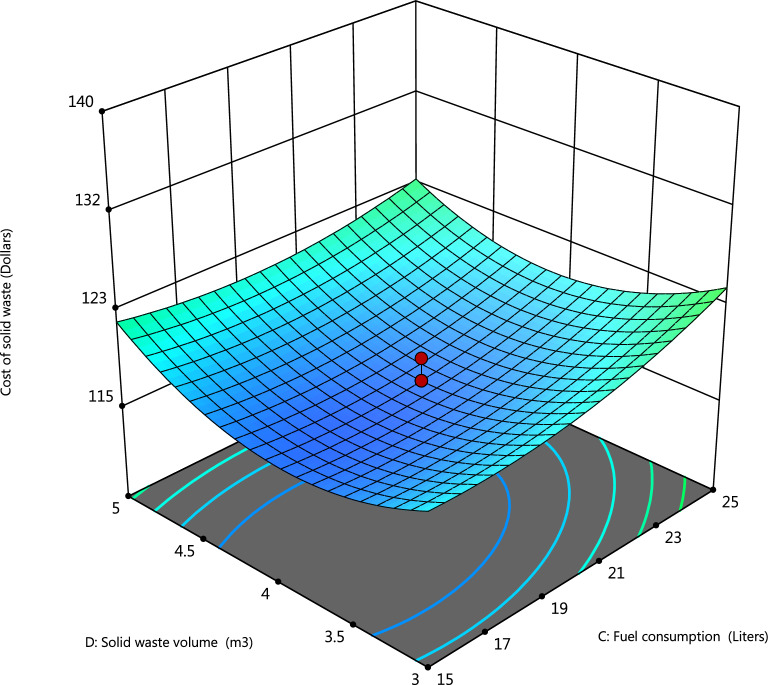
3D surface plots showing the dependency of the volume of solid waste collected (m^3^) and Fuel consumed in liters during collection on the cost of the solid waste management system (Design-Expert software v. 12).

The 3D surface model is shown in Figs. 3, and 4 was developed using the RSM modeling technique, thereby optimizing the design space and influencing each variable on the cost function. It is prominent to see the volume of waste collected, and fuel consumption has a greater influence on the cost of waste collected during each trip. In the numerical optimization phase, the system optimized the overall cost of waste collected in order to optimize the nos. of trips, workers involved, the volume of waste collected, and fuel consumption, as shown in Supplementary Fig. 3.

During this study, the design expert software was involved in the optimization of process parameters during the collection of MSW from Jeddah city. The use of design experiments and numerical optimization is quite effective in optimizing the different process parameters on the overall cost. The model is quite fit as the coefficient of determination (R^2^) was observed to be 1.000. Table 2 shows the optimized values along with observed values for the system.

## Results and discussion

### Characterization analysis of MSW in Saudi Arabia

The massive growth and management of MSW is a significant environmental problem for developing countries and the world. This is partly because of the inadequacy of effective treatment technologies for developing countries like India and suitable solutions. Therefore, baseline data on municipal solid should be developed, including generation and characterization analysis, leading to an improved MSW management system. As summarized from the existing performance of management of MSW is very low, and one of the possible methods for its improvement would be to categorize the waste generated at the study locations. Thus, it is mandatory to categorize the waste characterization. The organic waste fraction in MSW, particularly in Indian cities, is higher than the waste in developed countries. In this sense, environmentally-friendly facilities are of immediate importance in India for treating waste. In addition, the study areas in Saudi Arabia comply with a particular characteristic waste production pattern since the population is wide-ranging, resulting in the creation of dumpsites, and these specific places experience extensive temperature fluctuations and challenging terrain conditions, which affect the implementation of appropriate MSW management strategies. In the other tourist areas, the research regions often misinterpret the total volume and waste character. Therefore, the MSW generated in the study areas is generally classified as mixed waste without any distinctive classification. Carrying out a practical, economic, and environmentally sustainable waste management system is a significant factor. Furthermore, waste physical/chemical characterization will help politicians and community organizers minimize waste sites and recycle schemes for start-ups.

### Physical characterization

Physical characterization of waste involves classifying samples as biodegradable waste, non-biodegradable content, recyclable and inert materials into different components. The samples were divided into different components, including compostable, paper, plastic, textile, metal, rubber, etc. Finally, to determine physical characterization, the weight of every part was acquired then the samples were transmitted to the laboratory immediately for the analysis of moisture content. In addition, assessing the density of waste is crucial if an efficient waste management system is built. In developing engineered waste sites, density plays an important role. Density changes may occur when weather vibration in transport vehicles moves from a source to a dumping site due to handling, wetting, and drying.

### Chemical characterization

The chemical characterization of waste, including the close and final analysis and a fraction of MSW components and ash content, is carried out. The sample was prepared in compliance with ASTM. Other parameters such as humidity content, volatility material, ash content, solid carbon, and elementary analysis, include carbon, hydrogen, nitrogen, sulfur, and oxygen, have also been measured (C, H, N, S, and O, respectively). However, through the use of the bombs in the lab, the gross calorific value was determined as shown in Supplementary Figs. 4 and 5. The proportion of organic fractions in Jeddah and Madina was considered slightly higher, as shown in Supplementary Figs. 4 and 5. The difference in the seasonal physical composition of MSW indicates that the biodegradable waste fraction is higher in summer. The high temperature and the intake of additional materials, fruits and vegetable products during the summer season are less in winter, as shown in Supplementary Fig. 5. In the summer season, an influx of visitors can also contribute to a rise in waste during the summer season. In contrast to research locations in Saudi Arabia, however, the literature studies have shown a lower proportion of organic waste, which includes in Indian towns such as Jalandhar (34%), Varanasi (32%), Bhopal (41%).

Saudi Arabia plans to reduce its dependency on oil to generate energy, similar to many other nations. Yet Saudi Arabia's population growth rises enormously, hitting an average of 3.4 percent per annum and 80 percent urbanization (population living in cities). The progress of MSW has led to a substantial rise in population growth and urbanization. The estimated annual MSW of 14 million tonnes is currently produced in Saudi Arabia at 1.4 kg/day per capita, higher than the global average of 1.2 kg/day per capita. As shown in Supplementary Fig. 3, the total power generation of MSW is estimated for 2030. 2010 was selected as the basis for the forecasts. Supplementary Table 4 shows the waste composition of the Jeddah city with energy content in terms of kWh/kg. The total amount of waste generated during the period of 2016 to 2020 is shown in Supplementary Table 5 for a more compressive view on this topic. Supplementary Table 5 shown a comprehensive view of the waste generation as well as energy content for the said period. The research team has also taken an oral survey and field interviews under the supervision of experts and officers associated with this waste management process. The Eastern Province of Saudi Arabia has the largest area of about 672,522 km^2^ and is Saudi Arabia's third most populated province. In order to predict future waste production, 2010 was chosen as the base year. The per individual rate of waste is estimated to be 1.24 kg per day. The overall Eastern Saudi Province population is currently estimated to be 1.24 kg per capita. The total population of Eastern Saudi Arabia currently stands at 5.2 million (Supplementary Figs. 6 and 7). The population rise is expected to continue its 3.4 percent historical rate, which is Saudi Arabia's annual population growth. The MSW generation forecast results from 2010 to 2030 for the Eastern Province are illustrated in Supplementary Fig. 8. Supplementary Figure 8 shows that by the year 2030 (3545 thousand tonnes), a large quantity of waste will be produced, which requires careful management to mitigate long-term environmental and health impacts.

### Forecasting of WTE in Saudi Arabia

MSW processing and disposal in open sites is a current method for handling MSW in Saudi Arabia. In a few years, most sites are expected to surpass their capacity. The large volume of MSW generation and rich energy content show the ability to manage waste using WTE plants in Saudi Arabia. The existing waste dumping activity in Saudi Arabia involves processing unseparated waste in various residential and commercial areas for transportation into open waste dumps using a fleet of compressor waste trucks, each with a three-person crew (collection trips twice a day). While waste disposal in open waste sites is expected to exceed its capacity in a few years, land availability and relatively little capital are still the overall processes. Less than 15% of the collected MSWs in Saudi Arabia are currently recycled informally by scavengers who manually harvest paper, metals, and materials. Domestic oil consumption in Saudi Arabia is growing, representing over 25% of the country's output. As a result of population growth, domestic consumption is projected to rise by an annual percentage of 3.4%. This will increase infrastructure pressure and challenges current practices in the collection and disposal of MSW, especially in municipal services. The best approach is to optimize clean waste disposal and, at the same time, reduce the rate of waste production in residential areas (kill the problem before it happens). Saudi Arabia began introducing new technologies other than landfills in deserted areas for clean waste disposal.

### MSW ash utilization in concrete

MSW has been considered hazardous, and there is a dire need to reduce its volume. Incineration is a valuable technique to reduce the quantity of waste up to 70% by weight and 90% by volume. The incineration process of MSW generates electricity but leaves a considerable volume of ash known as MSW ash. This ash is generally disposed of in landfills, creating environmental problems and increasing landfill costs. Researchers are looking for ways to manage this ash as alternatives to open dumping and landfills. Based on its chemical properties, it was declared that MSW ash has an excellent potential to be utilized as a cementitious material in concrete production.

MSW ash is obtained from different locations, its chemical composition is evaluated, and the possibility of posing pozzolanic characteristics comparable to class-C as per ASTM C618 is identified. Si, Al, and Fe are the key indicators that represent the pozzolanic activity of ashes. If the combined sum of SiO_3_, Al_2_O_3_, and Fe_2_O_3_ is greater than 50%, MSW ash can be utilized in concrete to enhance its strength and durability. The properties of MSW ash indicate that it also contains high amounts of CaO, which could be beneficial in the hydration process of the cement paste in concrete. Besides that, it contains around 5.27% SO_3,_ which is allowable for pozzolanic ashes. Ash blending can provide a positive pozzolanic reaction. The chemical composition of the MSW ash could vary according to its type, but it contains high amounts of silica dioxide in almost all cases. The comparison of the chemical properties of MSW ash with ordinary Portland cement (OPC) as provided is shown in Supplementary Table 6.

The MSW analysis results are illustrated in Supplementary Fig. 9. Paper and cardboard waste included 17.09%, followed by 14.73% for food, 13.81% for wood, 11.41% for metals and 10.82% for metal, 7.87% for rubber, 3.13% for agricultural waste, and 6.81% for agricultural waste, Measurable small quantities of hazardous waste were found (0.63 percent ). The findings for the various zones of Al-Ahsa can be classified into five zones: A, B, C, D, and E. (Supplementary Fig. 10).

Repeating this two times may be necessary to detect new sensitization, especially in children when new symptoms appear. The recommended method of prick testing includes the appropriate use of specific allergen extracts, positive and negative controls. The test was done on the arm's volar surface, and the test can be done in the back. Many companies can synthesize allergens to be used in skin tests, and the results appeared after 15–20 min of application. The results will be positive if the wheel is equal to or larger than 3 mm in diameter. This study aimed to test some allergens from house dust mites, house dust, and fungi, collected from different areas of Jeddah city and compared their activities with that of the imported allergens.

In this research, 9 different allergens were isolated from Jeddah city, 2 species of house dust mite (Dermatophagoides farinae and Dermatophagoides pteronyssinus), house dust, and 6 samples of fungi including Candida albicans, Aspergillus fumigatus, Alternaria alternate, Penicillium chrysogenum, Cladosporium herbarum and Fusarium solani.

House Dust Mites gave a higher positive ratio in the sample, followed by dust, and the fungi were the lowest. The proportion of positive results of house dust mites in the prepared Saudi extracts was 50.6%, and the result was 59.1% for the Canadian extract. The percentage of positive results of the dust was 33.7% in the Saudi prepared extract and 26.5% in the Canadian extract; it is clear that the highest percentage was in the Saudi prepared extract. For fungi, the proportion of positive results in the Saudi prepared extract was 4.6% and 7.4% in Canadian extract, as shown in Supplementary Fig. 10. Each area represents a living area of the same quality. For instance, zone A is near the center, and MSW can contain large quantities of paper and cardboard waste. Zone D is a newly developed field, on the other hand. Therefore, as a product of construction company waste, it can contain large quantities of metals. This is explicit from Supplementary Fig. 15. In their MSW, Zones C and E are high in food.

### WTE scenario results

Three WTE scenarios, Mass Burn, recycled Mass Burn, and Biomethanation. Refuse-derived fuel (RDF) has been produced and analysed. The projected results are shown in Supplementary Figs. 7, 8 and 9 and in 2032 for Makkah, Madina, and Jeddah. Supplementary Figs. 9 to 11 show that in Makkah, Madina, and Jeddah, respectively, the Mass Burn scenario will yield approx. 87.0, 61.3, and 180.0 MW. Mass Burn's recycling power in Makkah, Madina, and Jeddah is 5.45, 3.84, and 11.25 MW, respectively. The RDF with the biomethane scenario can generate about 42.4, 29.9, and 87.3 MW respectively of the towns of Makkah, Madina, and Jeddah. In addition to the current development of solid waste management, the municipality can further enhance its efficiency by choosing resilient technology to achieve long-term sustainability targets to improve performance continuously (CPI). As seen in Supplementary Figs. Figs. 11 to 14, the fundamental concepts of CPI concentrate on continuous advances that can be the outcome of steady development or drastic technological change.

### New rules on waste management approved

To protect the environment and ensure public and citizen safety, Cabinet has approved new regulations on urban waste management in all cities and villages. The regulations would create an integrated system for MSW management. The Minister of Culture and Information told the Saudi press agency that waste was part of the separation, collecting, transport, storage/tripping, recycling, and treatment function after a weekly cabinet meeting observed over by Crown Prince Salman, deputy Prime Minister of Defense et al.-Salam Palace in Jeddah. The Ministry of Urban and Rural Affairs will supervise the operations and tasks of the MSW Management System. The plan is to protect citizens and the safety and protection of citizens. The Minister added that the Ministry must develop adequate programs to educate people about handling waste correctly. It has also called on the international community, in particular. The main suitable lessons derived from analysing global best practices are also enlisted in Supplementary Table 7.

## Future work

Solid waste includes rubbish, paper, plastics, metals, wood, and synthetics. Environmental authorities promote reuse to decrease waste, such as waste regeneration, recycling and composting, etc., for sustainability. Combustion is another solid waste method that contributed to reducing the required space. Solid waste burns at high temperatures in combustion plants, lowering waste volume and power generation. A significant impetus for encouraging solid waste reuse is Saudi vision 2030^41^. Every effort made to encourage garbage reuse must, however, be conducted within an overall framework that focuses on economic efficiency and environmental management. The following analysis of waste management in Saudi Arabia can help the Government to established feasible ways to adapt and improve waste management methods in a range of economic, regulatory and technological alternatives. This study is, nonetheless, relevant to other emerging nations, such as adjacent Golf Sister Countries, with comparable features.

The process of applying waste management strategies that might help to monitor important performance measurements then made accessible to municipal and industrial stakeholders should be engaged by local research organizations. Decisions on waste management should be taken together and in an inclusive manner, in accordance with all stakeholders' interests. The new law should be part of a waste reduction and management plan, establish long-term objectives and measures and support environmental management. Efforts should also be made to construct and modernize trash recycling plants. This is important for supporting a sustainable change in the usage of recycling materials in new building and infrastructure projects in Saudi Arabia. Future studies should also address how best practices are altered to better fit the local environment and cultural circumstances outside of Saudi Arabia. The efficiency of implementation of various waste management strategies may also be investigated. In general, this sort of effort needs to be accompanied by effective government programs (regulation, subsidies, and surveillance) to advance the Saudi objectives in the field of sustainability.

Further review of the financial, social, technological, and environmental aspects required by the authors is required to select the three scenarios mentioned in this document. The cost of capital per tonne, running costs, technical sophistication, labor levels, and geographical position would need to be analyzed to implement each of these scenarios. Based on the global trend in the successful implementation of these processes, their feasibility in KSA can be calculated. However, a specific scenario is essential and should be made based on the results of extensive analysis at the political level.

## Conclusion

The main conclusions and recommendations regarding future research are:

- MSW generated in SA contains about 40% organic waste, which indicates its potentiality to produce energy through anaerobic digestion.
- The abundant production of MSW increases the demand for landfills, which occupy vast areas of land and create environmental problems. Considering the energy production through MSW, incineration is the best solution, reducing the waste by 70% by weight and 90% by volume. The by-product of the incineration process known as MSW ash can be utilized in concrete production.
- MSW ash is characterized as pozzolanic material, which could positively influence the properties of concrete. New truck collection and the transfer were used to collect MSWs for the transfer stations and final landfill site from residential areas.
- There are inadequate small containers, many of which often vanish and have been used for other purposes by citizens.

The separation of sources is recommended since the separation of individual forms of waste into separately stored containers is recommended at the point of generation. The organic matter percentage in the MSW is minimal, so MSW composition is not advisable. Strict regulations and controls to avoid disposal of toxic waste by MSW should be enforced. The Municipality of Al-Ahsa is advised to run public awareness campaigns on the management and recycling of municipal waste.

In the three main cities across the Western Province of Saudi Arabia, this research assessed the possible contribution of WTE to meeting electricity needs and solved the problem of the depots. Three scenarios for Mass Burn, Mass Burn, and Recycling for WTE have been developed and analyzed Biomethanation for RDF. Up to 2032, the scenarios were expected. The research results show that over the other two scenarios Mass Burn Scenario has the highest potential. The three scenarios all offer a viable alternative for MSW disposal and are implemented may reduce the issue of landfills in the region.

## Supplementary Information

Supplementary Information.
