# Supplementary material for: Optimization of solid waste collection using RSM approach, and strategies delivering sustainable development goals (SDG’s) in Jeddah, Saudi Arabia
Source: Sci Rep. 2021 Aug 16;11:16612. doi: 10.1038/s41598-021-96210-0 (PMC8368206; doi:10.1038/s41598-021-96210-0)
Supplement: Supplementary file 1 — Supplementary Information. [file 41598_2021_96210_MOESM1_ESM.docx]

**Optimization of Solid waste collection using RSM approach, and strategies delivering sustainable development goals (SDG’s) in Jeddah, Saudi Arabia**

**Supplementary Figures and Supplementary Tables**

**
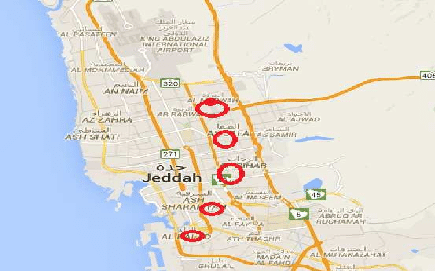
**

**Supplementary Figure-1 Location map and sampling zones (Satellite imagery source: The National Map;** [**http://viewer.nationalmap.gov/viewer/**](http://viewer.nationalmap.gov/viewer/)**).**

**
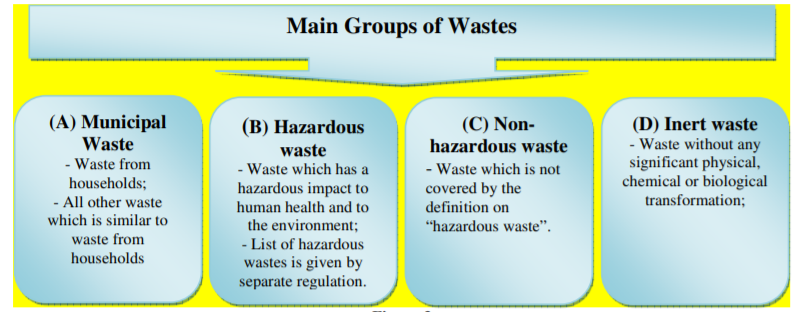
**

**Supplementary Figure-2 Main groups of Wastes.**

**
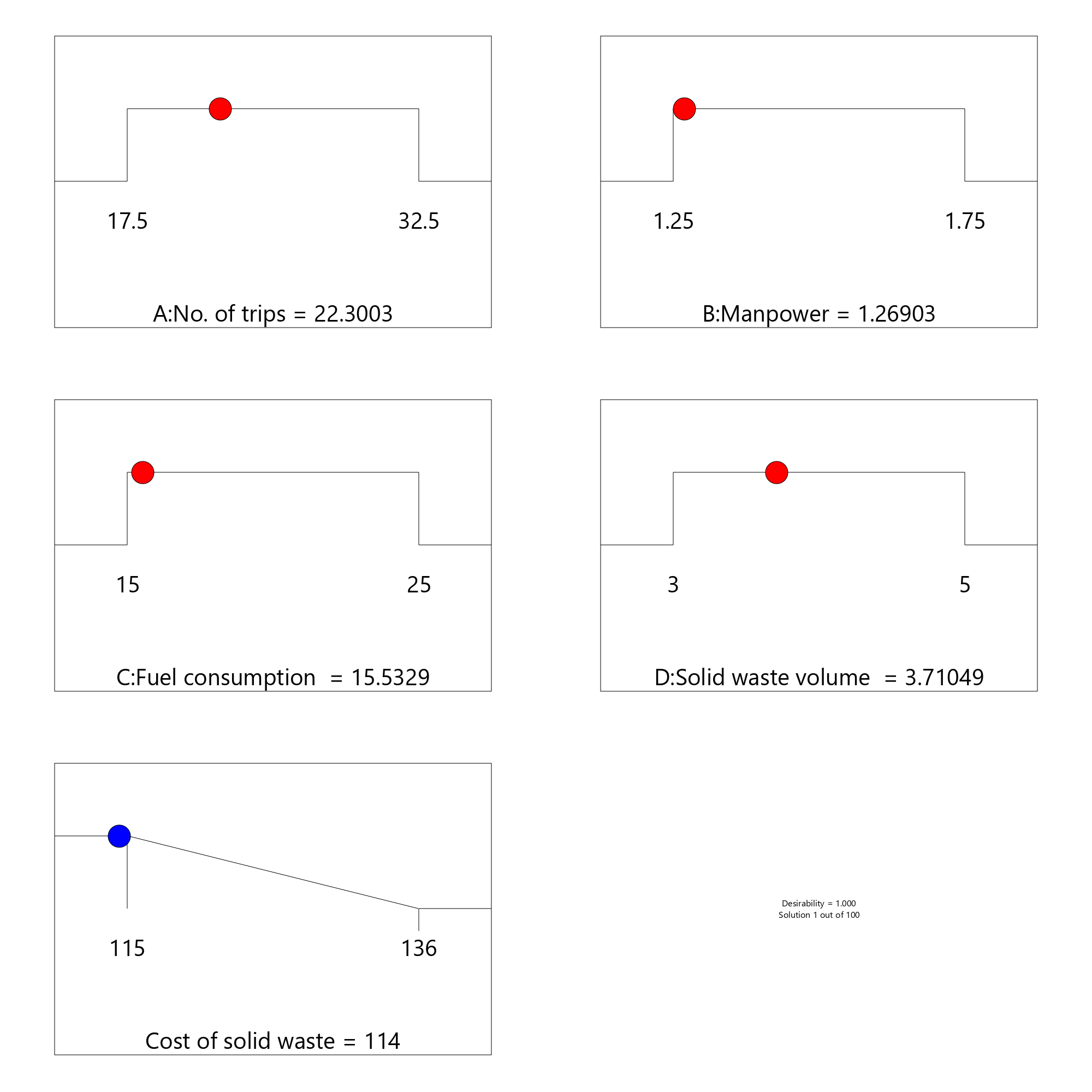
**

**Supplementary Figure 3: Optimization desirbaility the cost of solid waste management (Design Expert software v. 12).**

**Supplementary Figure 4: Pie chart showing the composition of MSW in Jeddah.**

**
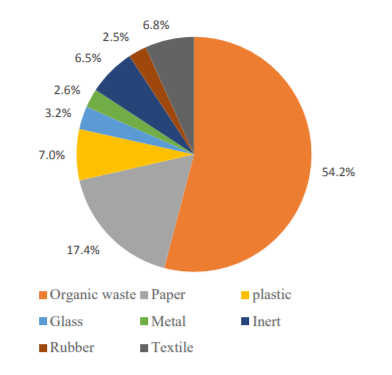

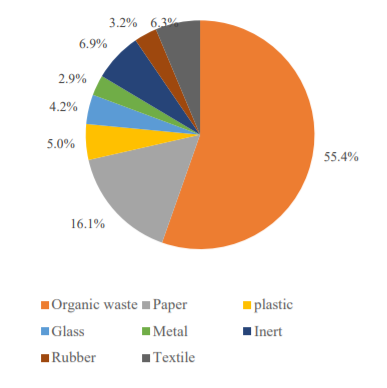
**

**Supplementary Figure 5: Pie chart showing the composition of MSW in Madina.**

**
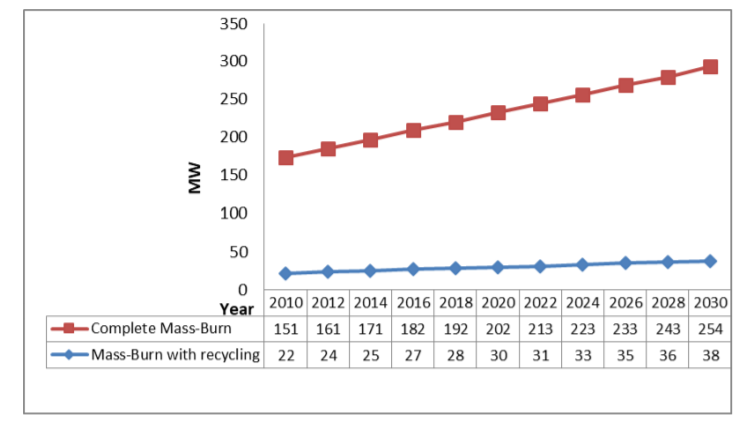
**

**
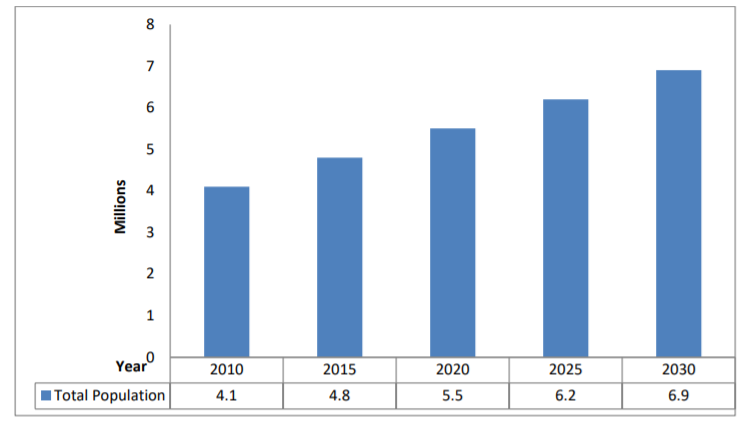
Supplementary Figure 6: For the years 2010–2030, Eastern Province has net power production capacity (MW).**

**Supplementary Figure 7. The population prediction results for Eastern Province.**

**
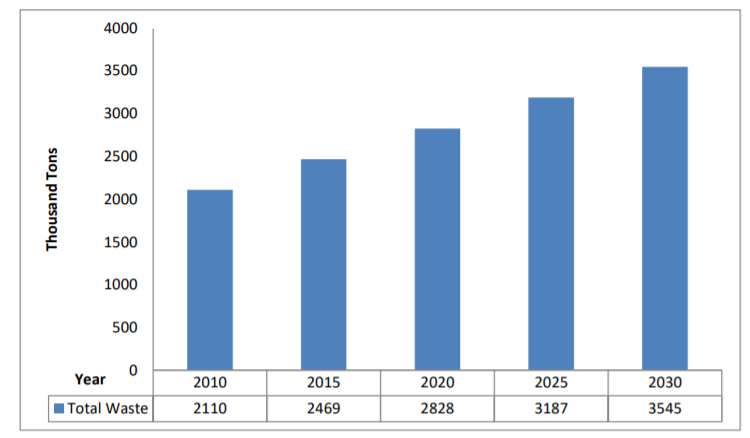
**

**Supplementary Figure 8. Forecast producing results in Eastern Province Municipal Solid Waste (MSW).**

**
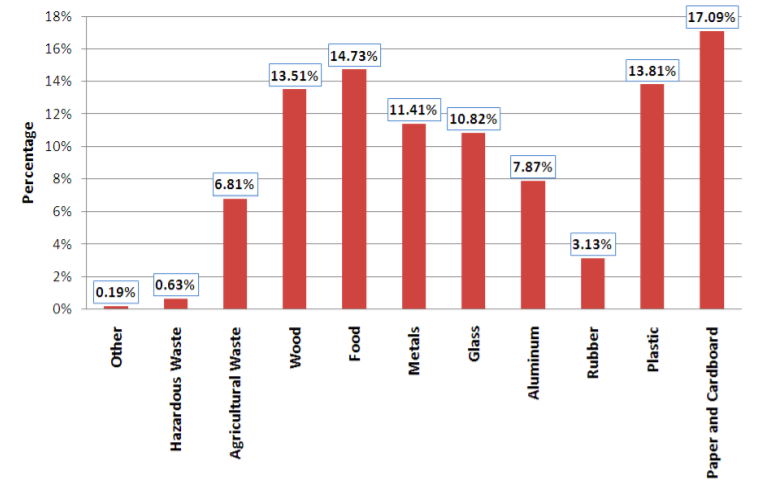
**

**Supplementary Figure 9. MSW percentage of material contents**

**
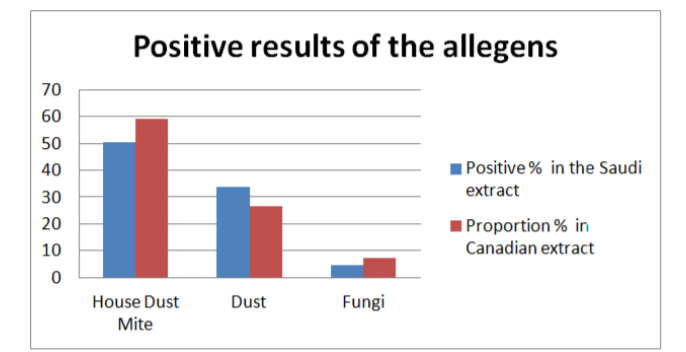
**

**Supplementary Figure 10. The positive results of the two tested extracts**

**
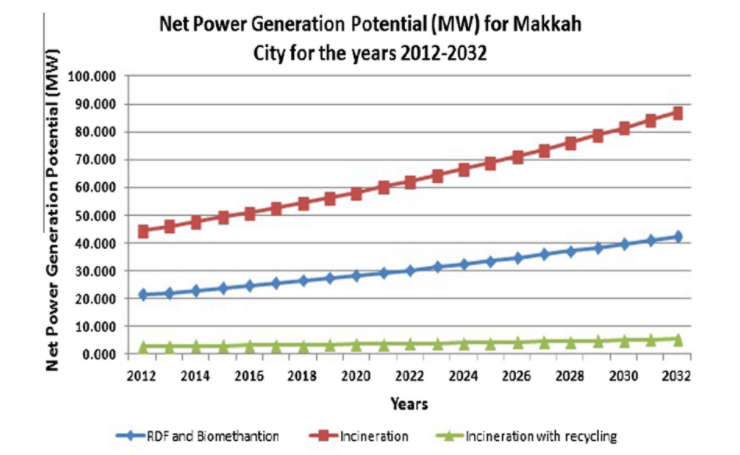
**

**Supplementary Figure 11. For the year 2012 to 2032, Makkah City has a net electricity generation capacity.**

**
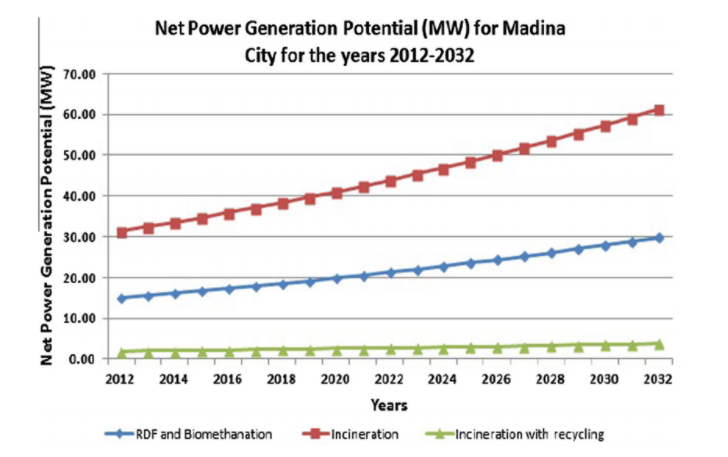
**

**Supplementary Figure. 12. For the years 2012–2032, Madina City has net power generation potential (MW).**

**
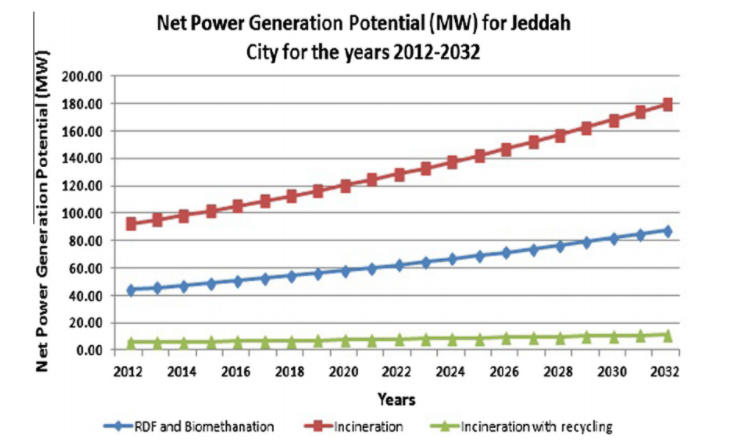
**

**Supplementary Figure. 13. For the years 2012–2032, Jeddah City has a net power generation potential.**

**
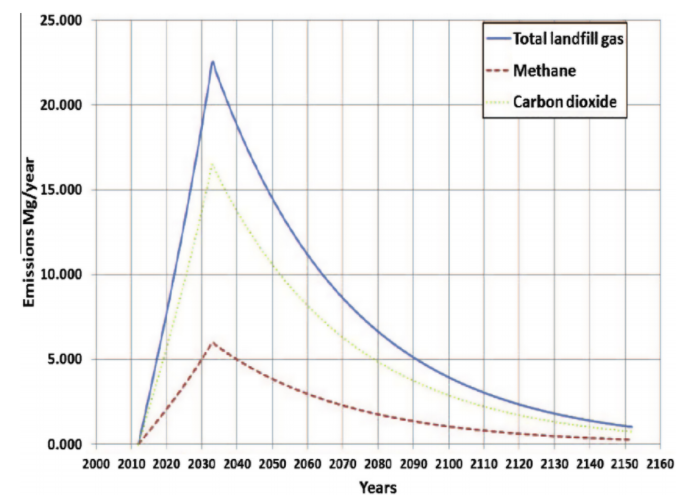
**

**Supplementary Figure. 14. RDF gas emission estimate for waste disposal at Madina site for 2012-2152 with biomethanation technology (Design expert v. 12).**


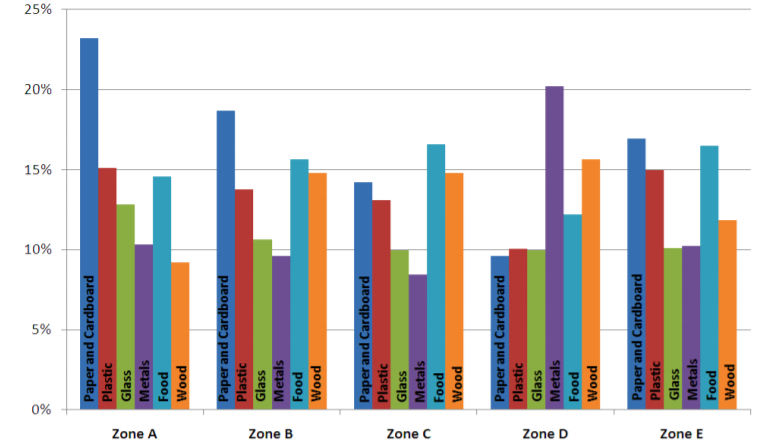


**Supplementary Figure. 15. Material percentages in Al-Ahsa areas**

**Supplementary Table 1 ANOVA analysis for Quadratic model**

| Source | Sum of Squares | df | Mean Square | F-value | p-value |  |
| --- | --- | --- | --- | --- | --- | --- |
| Model | 1083.12 | 14 | 77.37 | 61.89 | < 0.0001 | significant |
| A-No. of trips | 1.04 | 1 | 1.04 | 0.8333 | 0.3757 |  |
| B-Manpower | 35.04 | 1 | 35.04 | 28.03 | < 0.0001 |  |
| C-Fuel consumption | 63.37 | 1 | 63.37 | 50.70 | < 0.0001 |  |
| D-Solid waste volume | 3.37 | 1 | 3.37 | 2.70 | 0.1211 |  |
| AB | 85.56 | 1 | 85.56 | 68.45 | < 0.0001 |  |
| AC | 18.06 | 1 | 18.06 | 14.45 | 0.0017 |  |
| AD | 52.56 | 1 | 52.56 | 42.05 | < 0.0001 |  |
| BC | 203.06 | 1 | 203.06 | 162.45 | < 0.0001 |  |
| BD | 76.56 | 1 | 76.56 | 61.25 | < 0.0001 |  |
| CD | 14.06 | 1 | 14.06 | 11.25 | 0.0043 |  |
| A² | 85.00 | 1 | 85.00 | 68.00 | < 0.0001 |  |
| B² | 21.50 | 1 | 21.50 | 17.20 | 0.0009 |  |
| C² | 73.36 | 1 | 73.36 | 58.69 | < 0.0001 |  |
| D² | 469.07 | 1 | 469.07 | 375.26 | < 0.0001 |  |
| Residual | 18.75 | 15 | 1.25 |  |  |  |
| Lack of Fit | 12.75 | 10 | 1.27 | 1.06 | 0.5049 | not significant |
| Pure Error | 6.00 | 5 | 1.20 |  |  |  |
| Cor Total | 1101.87 | 29 |  |  |  |  |

**Supplementary Table 2 Fit statistics for Quadratic model**

| Std. Dev. | 1.12 |  | R² | 0.9830 |
| --- | --- | --- | --- | --- |
| Mean | **123.73** |  | **Adjusted R²** | **0.9671** |
| C.V. % | **0.9036** |  | **Predicted R²** | **0.9255** |
|  |  |  | **Adeq Precision** | **25.9834** |

**Supplementary Table 3 Coefficient under 95% confidence interval.**

| Vairables | Coefficient Estimate from paramters | df | Standard Error calculated | 95% CI Low | 95% CI High | VIF |
| --- | --- | --- | --- | --- | --- | --- |
| Intercept Value | 117.00 | 1 | 0.4564 | 116.03 | 117.97 |  |
| A-No. of trips | 0.2083 | 1 | 0.2282 | -0.2781 | 0.6948 | 1.0000 |
| B-Manpower | -1.21 | 1 | 0.2282 | -1.69 | -0.7219 | 1.0000 |
| C-Fuel consumption | 1.62 | 1 | 0.2282 | 1.14 | 2.11 | 1.0000 |
| D-Solid waste volume | 0.3750 | 1 | 0.2282 | -0.1114 | 0.8614 | 1.0000 |
| AB | -2.31 | 1 | 0.2795 | -2.91 | -1.72 | 1.0000 |
| AC | -1.06 | 1 | 0.2795 | -1.66 | -0.4667 | 1.0000 |
| AD | 1.81 | 1 | 0.2795 | 1.22 | 2.41 | 1.0000 |
| BC | -3.56 | 1 | 0.2795 | -4.16 | -2.97 | 1.0000 |
| BD | -2.19 | 1 | 0.2795 | -2.78 | -1.59 | 1.0000 |
| CD | -0.9375 | 1 | 0.2795 | -1.53 | -0.3417 | 1.0000 |
| A² | 1.76 | 1 | 0.2135 | 1.31 | 2.22 | 1.05 |
| B² | 0.8854 | 1 | 0.2135 | 0.4304 | 1.34 | 1.05 |
| C² | 1.64 | 1 | 0.2135 | 1.18 | 2.09 | 1.05 |
| D² | 4.14 | 1 | 0.2135 | 3.68 | 4.59 | 1.05 |

**Supplementary Table 4. Saudi Arabia’s MSW energy contents.**

| Solid waste type | Solid Waste Composition (%) | Energy Content available in solid waste (kWh/kg) | Energy Content in Waste (kWh/kg) LHV |
| --- | --- | --- | --- |
| Paper | 20.7 | 5.35 | 1.20 |
| Plastic | 6.5 | 7.54 | 0.56 |
| Glass | 5.8 | 0.00 | 0.00 |
| Wood | 12 | 3.98 | 0.27 |
| Textiles | 8.2 | 4.9 | 0.19 |
| Organic | 26.3 | 2.45 | 0.11 |
| Others | 20.5 | 4.06 | 0.21 |
| Total energy for mass burn with recycling (kWh/kg) | | | 0.38 |
| Total energy contents of complete mass burn (kWh/kg) | | | 2.51 |

**Supplementary Table 5: Data of total waste for the years 2016, 2017, 2018, and 2020.**

| S. No | Type of waste | Amount in tones | | | | Remark |
| --- | --- | --- | --- | --- | --- | --- |
|  |  | 2016 | 2017 | 2018 | 2020 |  |
| 1 | The amount of MSW incoming to the landfill | 5,956,734 | 1,956,734 | 1,856,182 | 1,976,834 |  |
| 2 | The number of dams that were received in the landfill | 4825006 | 2825006 | 2,373,391 | 2,915,321 |  |
| 3 | The number of tires that were received in the landfill | 10,322 | 8322 | 7,987 | 8,386 |  |
| 4 | The amount of plant waste contained in the landfill | 723684 | 523684 | 498,741 | 528,665 |  |
| 5 | The amount of waste received without being treated by the waste sorting unit | 37519 | 17519 | 16,170 | 17,787 | Is the waste received and rejected by the waste sorting unit |
| 6 | The amount of waste treated on the belt of the screening unit | 10,69396 | 969396 | 934,230 | 976,422 |  |
| 7 | The amount of organic matter extracted and processed | 792834 | 492834 | 467,115 | 497,975 |  |
| 8 | The amount of materials destroyed by the paper incinerator | 782 | 382 | 370.5 | 382.4 |  |
| 9 | The amount of waste destroyed by burial | 9532 | 1102 | 1,029 | 1,117 |  |

**SUPPLEMENTARY TABLE 6. CHEMICAL PROPERTIES OF MSW**

| Sample | MSW Ash | OPC |
| --- | --- | --- |
| Chemical Content | % | % |
| Silica dioxide (SiO_2_) | 53.16 | 20.61 |
| Aluminium trioxide (Al_2_O_3_) | 3.98 | 3.95 |
| Ferric oxide (Fe_2_O_3_) | 1.40 | 3.46 |
| Calcium oxide (CaO) | 19.13 | 63.95 |
| Titanium dioxide (TiO_2_) | 0.18 | 0.20 |
| Potassium oxide (K_2_O) | 0.94 | - |
| Magnesium oxide (MgO) | 1.49 | 1.93 |
| Phosphorus pentoxide (P_2_O_5_) | 5.00 | - |
| Sulfur trioxide (SO_3_) | 5.27 | 3.62 |
| Zirconium dioxide (ZrO_2_) | 0.67 | - |
| Sodium superoxide (NaO_2_) | 0.67 | - |

**Supplementary Table 7: Lessons derived from analysing global best practices.**

| Focus Areas of Global Best Practices | Examples of Typical Initiatives. | KSA Performance |
| --- | --- | --- |
| Conservation of Natural Habitats and Biological Species | Expansion and management of the natural reserves, conservation of the natural habitats and biological species outside the natural reserves through propagation and reintroduction of the endangered species. | Poor |
| Sustainable Management of Resources | Promotion of eco-tourism and the activities thereof, including water sports facilities, desert resorts, and etc. | Poor |
| Pollution Prevention and Environmental Compliance Monitoring | Reinforcement of the environmental monitoring means in terms of terrestrial, marine and coastal environments; monitoring the developmental activities, sources of pollution; and setting the necessary pressure mitigation plans. | Poor |
| Rehabilitation of Degraded Locations | Rehabilitation of the locations deteriorated as a result of the developmental activities and natural disasters through natural habitats reconstruction, coral reefs rehabilitation, and etc. | Poor |
| Climate Change Adaptation | Assessment of the impacts of climate change on ecosystems (coastal vulnerability index, etc.) and development of action plans for adaptation to climate change and mitigate the consequences thereof. | Poor |
